# Hypoglycemia Is Associated With Worse Outcomes in Patients With Cholangitis Despite Undergoing Endoscopic Retrograde Cholangiopancreatography

**DOI:** 10.7759/cureus.26964

**Published:** 2022-07-18

**Authors:** Adham E Obeidat, Ratib Mahfouz, Kevin Benavente, Landon A Kozai, Mahmoud M Mansour, Mohammad Darweesh, Nikolaos T Pyrsopoulos

**Affiliations:** 1 Hepatology, Rutgers University New Jersey Medical School, Newark, USA; 2 Internal Medicine, Kent Hospital/Brown University, Warwick, USA; 3 Internal Medicine, University of Hawaii, Honolulu, USA; 4 Internal Medicine, University of Missouri School of Medicine, Columbia, USA; 5 Internal Medicine, East Tennessee State University, Johnson City, USA; 6 Gastroenterology and Hepatology, Rutgers University New Jersey Medical School, Newark, USA; 7 Gastroenterology and Hepatology, Rutgers University, Newark, USA

**Keywords:** advanced endoscopy, mortality, hypoglycemia, ercp, acute cholangitis

## Abstract

Background

Hypoglycemia has been associated with poorer outcomes in hospitalized patients undergoing surgical interventions. In cholangitis, endoscopic retrograde cholangiopancreatography (ERCP) is often a critical adjunct to surgery, capable of diagnosing and treating various biliary and pancreatic pathologies. While technically less invasive than surgery, the effect of hypoglycemia on clinical outcomes of patients with cholangitis undergoing ERCP has not been elucidated.

Methodology

Data were extracted from the National Inpatient Sample (NIS) database from 2016 to 2019. Using the International Classification of Diseases, Tenth Revision, Clinical Modification (ICD-10-CM) codes, patients diagnosed with cholangitis and underwent ERCP were identified. Baseline demographic data, comorbidities, in-hospital mortality, hospital charges, and hospital length of stay (LOS) were extracted and compared based on the presence or absence of hypoglycemia. Statistical analysis was done using t-test and chi-square analyses. A multivariate analysis for the mortality odds ratio (OR) was calculated to adjust for possible confounders.

Results

A total of 256,540 patients with cholangitis who underwent ERCP were identified, and 2,810 of them had hypoglycemia during their hospitalization. The mean age of the hypoglycemia group was 64.41 years. Most patients were females (54%) and whites (57%). More patients in the hypoglycemia group had a history of alcoholism and congestive heart failure (CHF). Hypoglycemia was associated with higher odds of in-hospital mortality (OR = 6.71, confidence interval (CI) = 5.49-8.2; p < 0.0001). In addition to hypoglycemia, age >65 years, non-white race, and CHF were independently associated with higher mortality. Moreover, patients with hypoglycemia had higher total hospital charges ($87,147 vs. $133,400; p < 0.0001) and a significant increase in the LOS (9.7 vs. 6.7 days; p < 0.0001).

Conclusions

Previous studies in the surgical literature have linked hypoglycemia to increased incidence of atrial fibrillation, usage of mechanical ventilation, and application of circulatory support. Hypoglycemia may also affect the metabolism of the heart, leading to myocardial ischemia and malignant arrhythmias. However, it is unclear if hypoglycemia represents a proxy for the severity of patient illness as septic shock and renal insufficiency are common etiologies that may strongly impact mortality. Therefore, careful glycemic control during hospitalization should be practiced as hypoglycemia serves as a poor prognostic indicator that should not be overlooked.

## Introduction

Ascending cholangitis is an infection of the biliary system commonly caused by ductal obstruction. It classically manifests as Charcot’s triad of fever, jaundice, and right upper quadrant abdominal pain [1]. The mainstay of therapy includes alleviation of the biliary obstruction by endoscopic retrograde cholangiopancreatography (ERCP) [1]. Mortality rates of ascending cholangitis remain high despite therapeutic advances owing to the rapid progression of biliary infection to septicemia. Thus, the identification of risk factors and prognostic indicators in the management of this disease is important for patient optimization and survival [1,2].

Hypoglycemia has been found to be a poor prognostic indicator in previous studies. It is independently associated with higher mortality in the setting of sepsis and may indicate early sepsis and in-hospital deterioration in some clinical scenarios [3-5]. Prior retrospective studies have suggested increased mortality in patients with chronic liver disease who develop in-hospital hypoglycemia [6]. Patients undergoing surgery also face higher mortality when managed with stringent perioperative glycemic control [7,8]. Although less invasive than surgery, ERCP can potentially share some aspects of this risk profile. Thus, we seek to contribute to the periprocedural risk stratification of this patient population and clarify the effect of hypoglycemia in patients with ascending cholangitis undergoing ERCP.

An abstract of this study was submitted to the American College of Gastroenterology (ACG) Annual Meeting. Charlotte/North Carolina, October 21-26, 2022.

## Materials and methods

Data source

This is a retrospective study of patients admitted to a hospital in the United States between 2016 and 2019. The data were extracted from the Healthcare Cost and Utilization Project National Inpatient Sample (NIS) database. Because this study was done using a publicly available de-identified dataset (NIS), Institutional Review Board approval was deemed unnecessary. A 20% probability sample was collected and weighted to ensure that the selected population was nationally representative. Each admission in the database was assigned one diagnosis, up to 40 secondary diagnoses, and 25 procedures. These variables were defined via the International Classification of Disease, Tenth Revision, and Clinical Modification (ICD-10-CM) codes.

Study variables

**Figure 1:**
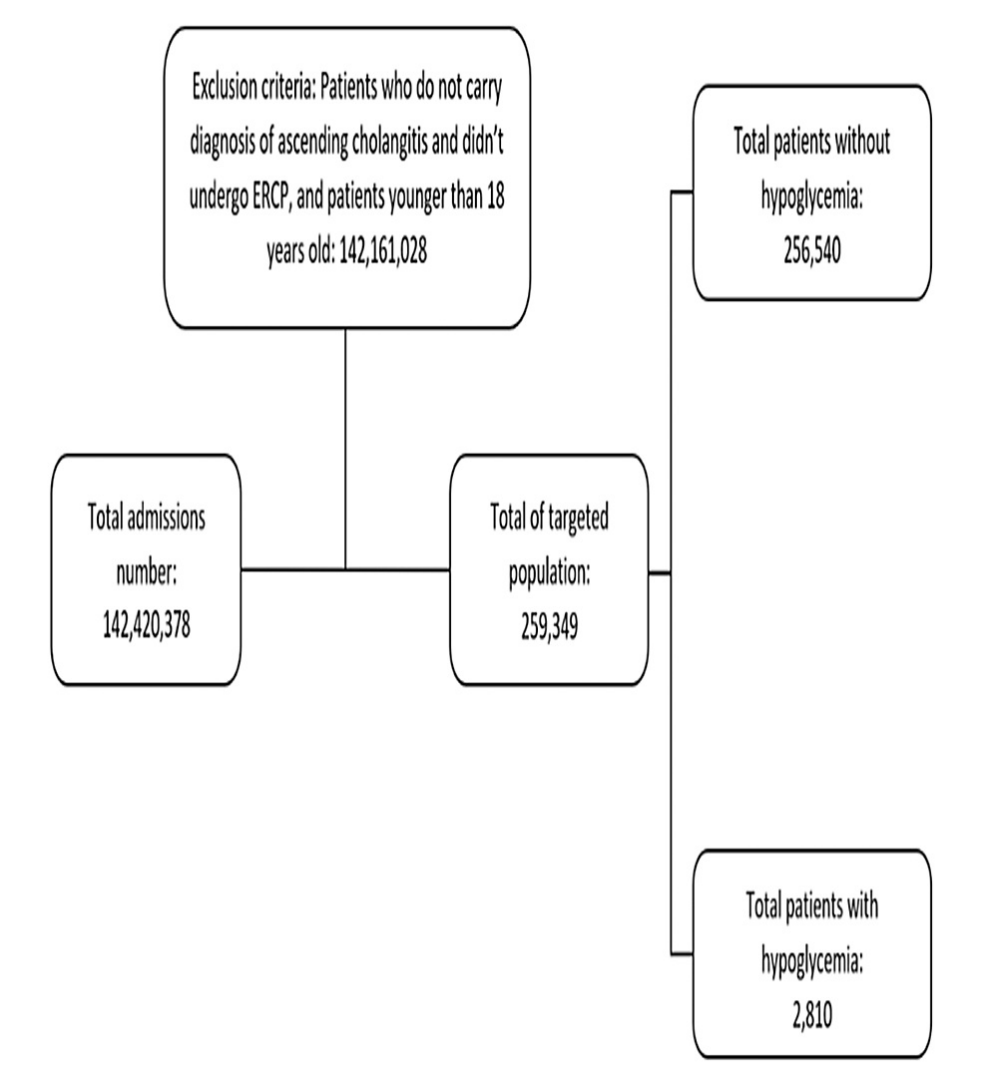
Flowchart of patients after applying inclusion and exclusion criteria.

Using ICD-10-CM codes, we identified patients >18 years old, who carried a diagnosis of ascending cholangitis, underwent ERCP, and had a diagnosis of hypoglycemia during admission. Patient’s age (in years), gender, and race (white, black, Hispanic, others) were collected and considered as baseline characteristics. The comorbidities included alcoholism, diabetes mellitus (DM), hypertension (HTN), congestive heart failure (CHF), smoking, and obesity (body mass index (BMI) >24.9 kg/m^2^) (Figure 1).

Statistical analysis

The statistical analysis was done using STATA software, version 17.0 (StataCorp., College Station, TX, USA). The characteristics of patients with cholangitis alone and those who had both cholangitis and hypoglycemia were described using descriptive statistics. In this study, multivariate logistic regression analysis was performed to determine factors associated with in-hospital mortality. Variables that were not statistically significant (p > 0.05) on univariate analysis were excluded from the multivariate analysis. The odds ratio (OR) at a 95% confidence interval (CI) was used to describe the association between the study and outcome variables. Statistical significance was defined as a two-tailed p-value of <0.05.

## Results

Demographics and clinical characteristics

Of the 259,349 patients who were admitted with cholangitis and underwent ERCP, 2,810 (1.08%) were diagnosed with concurrent hypoglycemia during admission (Figure 1). The mean age of patients with cholangitis and concurrent hypoglycemia was slightly less than patients without hypoglycemia (64.4 vs. 66 years, p = 0.02). The female gender was more prevalent in the hypoglycemia group (53% vs. 47%, p < 0.004). Moreover, African American race was more prevalent in the hypoglycemia group (21.5% vs. 8.8%, p < 0.001) while the white race was less prevalent (57% vs. 66%, p < 0.004). More patients with alcoholism and CHF were found in the hypoglycemia group. Interestingly, DM, HTN, and obesity were more common in the non-hypoglycemic group (Table 1).

**Table 1:** Demographic and clinical characteristics of patients with cholangitis and concurrent hypoglycemia versus cholangitis without hypoglycemia. DM: diabetes mellitus; HTN: hypertension; CHF: congestive heart failure

Variable	No hypoglycemia	Hypoglycemia	P-value
Age (mean, year)	66	64.4	0.02
Gender N (%)			<0.004
Male	134,426 (52.4%)	1,292 (46%)	
Female	120,573 (47%)	1,517 (54%)	
Race N (%)			<0.001
White	170,060 (66.29%)	1,610 (57.3%)	
African American	22,806 (8.89%)	605 (21.53%)	
Hispanic	30,400 (11.85%)	240 (8.54%)	
Others	33,299 (12.98%)	355 (12.63%)	
Comorbidities (%)			
Alcoholism	12,134 (4.73%)	275 (9.79%)	<0.001
DM	79,039 (30.81%)	85 (3.02%)	<0.001
HTN	156,540 (61.02%)	1,489 (53.02%)	<0.001
CHF	34,042 (13.27%)	455 (16.19%)	0.044
Smoking	80,963 (31.56%)	890 (31.67%)	0.955
Obesity	37,893 (14.75%)	260 (9.25%)	<0.001

Inpatient outcomes

In-hospital mortality was significantly higher in the hypoglycemia group compared to the non-hypoglycemia group (26.56% vs. 5%, p < 0.001). Total hospital charges were significantly higher in the hypoglycemia group compared to the non-hypoglycemic group ($133,400 vs. $87,147, p < 0.001). Moreover, the mean length of stay (LOS) was significantly higher in the hypoglycemia group (9.7 vs. 6.7 days, p < 0.001). Table 2 summarizes these findings.

**Table 2:** Comparison of in-hospital outcomes between patients with cholangitis and hypoglycemia versus patients with cholangitis without hypoglycemia.

Outcome	No hypoglycemia	Hypoglycemia	P-value
Total hospital charge ($)	87,147	133,400	<0.001
Length of stay (days)	6.7	9.7	<0.001
In-hospital mortality (%)	5%	26.56%	<0.001

In-hospital mortality

**Figure 2:**
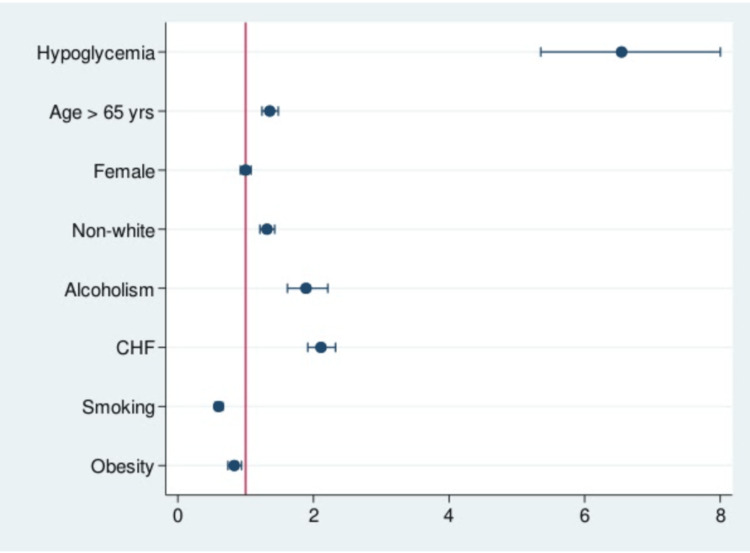
Odds ratio plot chart of in-hospital mortality in patients with cholangitis who underwent ERCP. CHF: congestive heart failure; ERCP: endoscopic retrograde cholangiopancreatography

As stated above, the prevalence of in-hospital mortality in patients who were admitted with cholangitis and underwent ERCP with concurrent hypoglycemia was higher than the non-hypoglycemia group (Table 2). This was also reflected in the multivariate analysis (Table 3). Patients in the hypoglycemia group had higher odds of dying during hospitalization (OR = 6.71, 95% CI = 5.49-8.2; p < 0.0001). Moreover, age >65 years (OR = 1.35, 95% CI = 1.24-1.48; p < 0.0001), non-white race (OR = 1.31, 95% CI = 1.21-1.42; p < 0.0001), alcoholism (OR = 1.89, 95% CI = 1.61-2.21; p < 0.0001), and CHF (OR = 2.11, 95% CI = 1.91-2.32; p < 0001) were all associated with increased chance of in-hospital mortality. Interestingly, obesity (OR = 0.83, 95% CI = 0.73-0.93; p = 0.003) and smoking (OR = 0.6, 95% CI = 0.54-0.66; p < 0.0001) were associated with significant decrease in mortality (Table 3) (Figure 2).

**Table 3:** Univariate and multivariate analyses of potential factors that affect the in-hospital mortality in patients with cholangitis who underwent ERCP. OR: odds ratio; CI: confidence interval; DM: diabetes mellitus; HTN: hypertension; CHF: congestive heart failure; ERCP: endoscopic retrograde cholangiopancreatography

In-hospital mortality	OR (95% CI)	P-value	aOR (95% CI)	P-value
Hypoglycemia	6.74 (5.56-8.17)	<0.0001	6.54 (5.35-8)	<0.0001
Age >65 years	1.41 (1.3-1.54)	<0.0001	1.35 (1.24-1.48)	<0.0001
Female	1.02 (0.95-1.11)	0.463	0.99 (0.92-1.07)	0.968
Non-white	1.29 (1.19-1.4)	<0.0001	1.31 (1.21-1.42)	<0.0001
Alcoholism	1.58 (1.36-1.849	<0.0001	1.89 (1.61-2.21)	<0.0001
DM	0.98 (0.9-1.07)	0.736	-	-
HTN	0.93 (0.86-1.01)	0.089	-	-
CHF	2.21 (2.02-2.42)	<0.0001	2.11 (1.91 - 2.32)	<0.0001
Smoking	0.6 (0.54-0.66)	<0.0001	0.6 (0.54 -0.66)	<0.0001
Obesity (BMI >24.9 kg/m^2^)	0.81 (0.72-0.92)	0.001	0.83 (0.73 - 0.93)	0.003

## Discussion

Our study suggests that in patients with cholangitis, hypoglycemia was associated with increased mortality despite undergoing ERCP. A possible explanation for these results includes an increase in cardiovascular complications associated with the combination of hypoglycemia and endoscopy. Low serum glucose levels affect the metabolism of the heart, leading to an increased frequency of myocardial ischemia [9]. The sympathoadrenal-mediated response to hypoglycemia leads to catecholamine release, which when compounded by the physiologic stressors of endoscopy, including the reduction in systemic vascular resistance and cardiac output induced by anesthesia, potential hypoxia, as well as pain and sympathetic activation, hemodynamic stability may be severely compromised [10]. Previous studies have linked perioperative hypoglycemia in the surgical literature to increased incidence of malignant arrhythmias, usage of mechanical ventilation, and need for circulatory support [11]. A large portion of our patient population carried a diagnosis of heart failure as well. This is a potential confounder and limitation of our study as hypoglycemia may have a more pronounced and morbid effect on these patients, in addition to an already elevated sepsis mortality at baseline compared to non-heart failure patients [12]. A large portion of the hypoglycemic cohort also abused alcohol, which is another independent risk factor increasing mortality in septic patients [13].

Even in the absence of surgical or procedural intervention, hypoglycemia during hospital admission represents a prognostic indicator of increased mortality in sepsis [5]. This is further supported by studies examining the influence of inpatient glycemic control on critically ill patients, in which an intensive strategy targeting serum glucose ranging from 81 mg/dL to 108 mg/dL was associated with increased mortality when compared to a more liberal strategy [14]. It is reasonable to extrapolate this data to patients with cholangitis who frequently present with sepsis. However, unlike other more common etiologies of sepsis that typically do not require invasive procedures for their management, such as pneumonia or urinary tract infections (UTI), the impetus to avoid hypoglycemia in cholangitis patients should be emphasized in anticipation of ERCP.

The high mortality rate demonstrated among the hypoglycemic cohort is consistent with mortality reported in previous studies examining severe cholangitis [15,16]. However, the most current 2018 Tokyo Guidelines for the grading of cholangitis do not include hypoglycemia as a part of the prognostic criteria [2]. Given the high mortality we present in this subgroup, a low threshold to classify patients presenting with hypoglycemia as having severe cholangitis should be maintained. Furthermore, urgent ERCP, within 24 hours of presentation, should be seriously considered in these patients as long as correction of the blood sugar can be achieved prior to the procedure. Further studies should be conducted to determine if hypoglycemia should be included as a prognostic indicator of severe cholangitis in future guidelines, as well as the ideal timing of ERCP in these patients.

Mechanistically, it is unclear if hypoglycemia itself is the causative driver for mortality, or if it is merely a proxy for the severity of the biliary infection. While causation cannot be determined given the retrospective design of this study, hypoglycemia in cholangitis should alert clinicians to the associated increased mortality in these patients and potentially guide the urgency of endoscopic intervention. Careful glycemic control and correction on admission and prior to ERCP should be practiced, with perioperative hypoglycemia serving as a poor prognostic indicator that should not be overlooked.

Our study has some limitations. Our conclusions may be confounded by the risk of repeat hospitalization as it is difficult to account for multiple admissions for one patient in the NIS. However, mortality rates are unlikely to be affected. Misclassification can happen due to under or over-coding, although the large number of patients strongly plays against substantial misclassification bias. NIS undergoes data quality assessment annually to ensure the internal validity of the data. Additionally, it can be hard to adjust for potential unmeasured confounders in observational studies which may affect our estimates for the reported associations among included covariates.

## Conclusions

In patients being treated for ascending cholangitis with ERCP, we demonstrate hypoglycemia to be a direct prognostic indicator associated with high mortality and an increased hospital LOS despite undergoing intervention with ERCP. This data could be applied as a potential adjunct to the 2018 Tokyo Guidelines severity assessment, particularly in clinical scenarios in which the severity of the disease or the stability of the patient remains unclear. By incorporating hypoglycemia as a prognostic risk modifier, attention toward more careful glycemic optimization and better-timed endoscopic interventions could potentially lead to better outcomes in patients with cholangitis.
